# Autoimmune Ocular Surface Disorders: From Molecular Immunopathogenesis to Regenerative and Surgical Therapeutics

**DOI:** 10.3390/cells15040378

**Published:** 2026-02-22

**Authors:** Wojciech Luboń, Marta Świerczyńska, Katarzyna Jadczyk-Sorek, Dorota Wyględowska-Promieńska

**Affiliations:** 1 Department of Ophthalmology, Faculty of Medical Sciences, Medical University of Silesia, 40-514 Katowice, Poland; katarzyna.jadczyk@sum.edu.pl (K.J.-S.); dwygledowska@sum.edu.pl (D.W.-P.); 2 Department of Ophthalmology, Professor K. Gibiński University Clinical Center, Medical University of Silesia, 40-514 Katowice, Poland; marta.swierczynska@sum.edu.pl; 3 Department of Clinical Genetics and Rare Diseases, Faculty of Medical Sciences in Katowice, Medical University of Silesia, 40-514 Katowice, Poland

**Keywords:** autoimmune ocular surface disease, systemic lupus erythematosus, primary Sjögren’s syndrome, ocular cicatricial pemphigoid, interferon signaling, mesenchymal stem cells, amniotic membrane transplantation, multi-omics profiling, precision immunotherapy, tear film biomarkers

## Abstract

**Highlights:**

**What are the main findings?**

- Autoimmune ocular surface diseases—particularly systemic lupus erythematosus, primary Sjögren’s syndrome, and ocular cicatricial pemphigoid—share convergent molecular pathways involving cytokine imbalance (IFN-I, IL-6, IL-17), epithelial–mesenchymal transition, and complement activation, resulting in chronic epithelial and adnexal injury.
- Recent therapeutic advances integrate biologic agents (belimumab, anifrolumab, and JAK inhibitors) with regenerative and surgical approaches such as mesenchymal stem cell transplantation, amniotic membrane grafting, and keratoprosthesis, establishing a new immunoregenerative paradigm in ocular surface therapy.

**What are the implications of the main findings?**

- Deciphering molecular immunopathogenesis enables precision-based therapy tailored to cytokine profiles and disease phenotypes, marking a transition from empirical immunosuppression to mechanism-driven intervention.
- The convergence of multi-omics analytics, artificial intelligence-based diagnostics, and regenerative medicine provides a translational framework for restoring immune homeostasis and achieving true ocular surface regeneration.

**Abstract:**

Autoimmune ocular surface diseases represent a complex group of disorders in which systemic immune dysregulation triggers chronic inflammation, epithelial dysfunction, and progressive tissue fibrosis. Systemic lupus erythematosus, primary Sjögren’s syndrome, and ocular cicatricial pemphigoid are the principal entities linking systemic autoimmunity to ocular surface pathology. These conditions share convergent mechanisms—including dysregulated cytokine signaling (IFN-I, IL-6, and IL-17), complement activation, and epithelial–mesenchymal transition—culminating in tear film instability and visual impairment. Recent advances in molecular immunology and omics profiling have elucidated disease-specific pathways and identified actionable therapeutic targets. Conventional immunosuppressants such as corticosteroids and cyclosporine remain fundamental, yet emerging biologics targeting BAFF, IFNAR, and JAK/STAT signaling—alongside regenerative strategies employing mesenchymal and induced pluripotent stem cells—are transforming disease management. Parallel innovations in amniotic membrane transplantation, keratoprosthesis, and bioengineered corneal scaffolds integrate structural reconstruction with immune modulation. Furthermore, the convergence of multi-omics analytics, artificial intelligence-assisted diagnostics, and microbiome-based immunomodulation heralds a new era of precision ophthalmology. This review synthesizes current molecular insights, clinical observations, and translational advances that collectively redefine autoimmune ocular surface diseases—from chronic inflammatory disorders into a targetable, regenerative, and potentially reversible spectrum of conditions.

## 1. Introduction

Autoimmune-mediated disorders of the ocular surface constitute a clinically and biologically heterogeneous group of conditions in which systemic immune dysregulation leads to localized epithelial injury, destabilizes the tear film, and promotes progressive tissue remodeling [1,2]. The ocular surface—comprising the cornea, conjunctiva, tear film, and adnexal glands—functions as a specialized mucosal interface that preserves optical clarity through tightly regulated epithelial, neural, and immune interactions under constant environmental stress [3,4]. Disruption of this interface by systemic autoimmunity transforms physiological immune surveillance into chronic inflammation, ultimately compromising visual function and quality of life [4,5,6,7].

Systemic lupus erythematosus (SLE), primary Sjögren’s syndrome (pSS), and ocular cicatricial pemphigoid (OCP) represent distinct autoimmune disease entities that converge on ocular surface pathology through different pathogenic architectures. SLE is characterized by systemic immune-complex-mediated inflammation, pSS primarily reflects a gland-centric autoimmune epithelitis, whereas OCP constitutes a localized mucous membrane-directed autoimmunity targeting the epithelial basement membrane zone [1,2,3]. Across these conditions, loss of immune tolerance initiates cytokine-driven inflammatory cascades that have been characterized primarily through clinical tear proteomics, conjunctival impression cytology, and transcriptomic profiling. In SLE, type I interferon-dominant signaling and immune-complex-mediated complement activation primarily affect the lacrimal and meibomian glands, whereas in pSS, Th17- and BAFF-driven inflammation predominates within lacrimal glandular tissue and the conjunctival epithelium. In OCP, local complement activation and TGF-β-mediated fibrotic signaling target the conjunctival basement membrane zone and stromal fibroblasts [4,8,9,10]. These signals converge on epithelial and glandular cells, impairing mucin production, destabilizing the tear film, and promoting epithelial apoptosis, disrupting the basement membrane, and driving extracellular matrix degradation mediated predominantly by MMP-9 activity [1,3,11,12]. Longitudinal clinical observations indicate that, rather than resolving, these inflammatory processes persist locally and may progress independently of systemic disease activity [6,7].

Recent clinical cohort studies, supported by tear proteomic analyses and glandular imaging, demonstrate that autoimmune ocular surface involvement is not merely a secondary consequence of sicca symptoms but reflects disease-specific immunopathogenic mechanisms [1,2,3]. In SLE, interferon-mediated epithelial stress and immune-complex deposition alter both lacrimal and meibomian gland function, even in the absence of secondary Sjögren’s syndrome [3]. In pSS, evidence derived from transcriptomic analyses of lacrimal gland biopsies and machine-learning-assisted gene-expression profiling indicates that Th17–BAFF-driven immune networks, together with activation of pyroptosis-related genes (including *NLRP3* and *GSDMD*), sustain glandular destruction and chronic conjunctival epithelial inflammation [9,10,13,14]. In contrast, OCP is dominated by complement activation and TGF-β-mediated epithelial–mesenchymal transition (EMT), driving progressive fibrosis and conjunctival scarring [6,9,13]. These shared yet distinct molecular pathways underscore the need for mechanism-oriented rather than symptom-based classification of autoimmune ocular surface disease [13,14].

In parallel with advances in immunopathogenesis, therapeutic strategies have expanded beyond empirical immunosuppression [5,15]. Targeted biologics modulating BAFF, interferon receptors, and JAK/STAT signaling, together with regenerative approaches such as mesenchymal stem cell therapy, platelet-rich plasma, and amniotic membrane transplantation, are redefining disease management [15,16,17,18,19]. However, significant gaps remain in translating molecular insights into personalized therapeutic algorithms, particularly regarding disease heterogeneity, biomarker-guided stratification, and long-term restoration of epithelial homeostasis [6,9,15].

However, despite the rapid expansion of molecular and clinical data, several critical gaps persist in the current literature [6,9,13,15]. The relative contribution of systemic immune activation versus local ocular surface-specific immune amplification remains incompletely defined across autoimmune entities, and it is still controversial whether autoimmune ocular surface disease represents a uniform inflammatory phenotype or a spectrum of mechanistically distinct endotypes requiring differential therapeutic strategies. Moreover, although numerous cytokine, complement, and regenerative pathways have been identified, their integration into a coherent, clinically actionable framework remains fragmented.

Accordingly, this review focuses in greater mechanistic depth on SLE, pSS, and OCP as prototypical autoimmune entities in which ocular surface involvement has been most extensively characterized at the molecular and cellular levels, while presenting other autoimmune conditions in a more illustrative context and synthesizing molecular, cellular, and translational evidence across diseases within an integrated pathogenic framework linking immune activation, epithelial dysfunction, fibrotic remodeling, and regenerative failure. Rather than providing a purely descriptive overview, it clarifies shared versus disease-specific immunopathogenic mechanisms, highlights unresolved controversies, and integrates emerging pharmacologic, regenerative, and surgical strategies into a unifying immunoregenerative model. By doing so, this work aims to reposition autoimmune ocular surface disease from a symptom-driven clinical entity toward a mechanism-based, stratifiable, and therapeutically targetable spectrum.

## 2. Molecular and Cellular Mechanisms of Autoimmune Ocular Surface Damage

The ocular surface represents a complex immunologic and neuroepithelial ecosystem, where epithelial, stromal, neural, and immune components operate in concert to sustain tissue integrity and optical clarity [4,10]. In autoimmune conditions affecting the ocular surface, immune dysregulation arises through disease-specific pathways that variably combine systemic immune activation, organ-specific autoimmunity, and localized mucosal immune responses. While SLE involves systemic cytokine and immune-complex signaling, pSS is dominated by lymphocytic infiltration of exocrine glands, and OCP reflects localized antibody- and complement-mediated injury at the mucosal basement membrane zone [1,5,8,11]. Deciphering the interaction between cytokine signaling, complement activation, oxidative stress, and epithelial–stromal crosstalk is essential for understanding how systemic immune activation translates into site-specific ocular surface pathology [5,8].

### 2.1. Immune Dysregulation and Cytokine Pathways

Immune dysregulation is a defining feature of autoimmune ocular surface disease and results from the loss of immune tolerance with persistent activation of T helper cell subsets, particularly Th1 and Th17 cells. Activated effector lymphocytes secrete cytokines such as IFN-γ, TNF-α, IL-17A, and IL-22, leading to altered epithelial metabolism, reduced mucin production, and destabilization of the tear film [1,2,4,10]. In parallel, elevated tear concentrations of IL-6, IL-1β, and IL-17 correlate with KCS severity and meibomian gland dropout [1,3,10]. At the tissue level, sustained cytokine exposure induces epithelial apoptosis and MMP-9 activation, promoting basement membrane degradation and increased epithelial permeability [4,11]. When persistently active, these inflammatory networks drive the transition from transient immune activation toward chronic epithelial injury [6].

Transcriptomic and single-cell analyses have highlighted the IL-18–Th17 axis as a pivotal regulatory node bridging systemic and local inflammation. IL-18 upregulates the transcription factor Bhlhe40, enhancing Th17 differentiation and amplifying IL-17-driven cytokine signaling in the ocular surface microenvironment [10]. Simultaneously, overexpression of B-cell activating factor (BAFF) supports the survival of autoreactive B cells and promotes the generation of pathogenic autoantibodies [9,10]. This dual activation of Th17 cells and B cells sustains a proinflammatory environment that can persist during periods of systemic disease quiescence and contribute to relapsing ocular surface inflammation.

### 2.2. Autoantibody and Complement Activation

Autoantibody-mediated complement activation is a central effector mechanism in autoimmune ocular surface injury. In SLE, immune complexes containing anti-dsDNA and anti-Ro/SSA antibodies deposit along epithelial and vascular interfaces, triggering classical complement activation [4,15]. The resulting generation of C3a and C5b-9 (membrane attack complex) induces epithelial cytotoxicity, endothelial dysfunction, and microvascular inflammation within conjunctival and corneal tissues [4,20]. Immunofluorescent studies have demonstrated linear or granular immunoglobulin deposition at the epithelial–stromal interface, paralleling cutaneous lupus and mucous membrane pemphigoid, confirming complement’s pivotal role in localized tissue damage [21].

Complement activation extends beyond direct cytolytic injury. C3a and C5a fragments serve as potent chemoattractants, promoting the recruitment of neutrophils and mononuclear cells and intensifying local inflammation [10,22]. Complement-derived signals further enhance T-cell activation and angiogenic factor release from ocular epithelial cells, sustaining chronic tissue remodeling. Crosstalk between complement and epithelial pathways can also induce fibroblast activation and epithelial–mesenchymal transition (EMT), establishing a mechanistic bridge between immune-mediated injury, angiogenesis, and fibrotic scarring [5,6,22]. The growing recognition of complement as a key effector mechanism has spurred interest in therapeutic inhibition of its terminal components—such as C5 blockade with eculizumab or C3 inhibition with avacincaptad pegol—as potential strategies to mitigate autoimmune ocular surface damage [22].

### 2.3. Oxidative Stress and Mitochondrial Dysfunction

#### 2.3.1. Redox Imbalance and Mitochondrial Stress in Autoimmune Ocular Disease

Oxidative stress has emerged as a recurrent feature associated with chronic inflammation and epithelial dysfunction in autoimmune ocular surface disease [10,11]. Experimental and translational studies suggest that proinflammatory cytokines, including IL-1β, TNF-α, and IFN-γ, may contribute to mitochondrial stress and redox imbalance in corneal and conjunctival epithelial cells; however, direct causal relationships between individual cytokines and mitochondrial dysfunction remain incompletely defined and appear to be context dependent [10,11,19]. Mitochondrial DNA damage, impaired ATP production, and epithelial apoptosis observed in autoimmune dry eye, therefore, likely reflect the convergence of inflammatory, metabolic, and oxidative signals rather than a single linear pathway [11,19].

#### 2.3.2. Disease-Specific Roles of Oxidative Stress and Antioxidant Modulation

In parallel, oxidative stress induces the overexpression of heat shock proteins (HSP70, HSP90), which act as autoantigens and amplify autoimmune signaling through molecular mimicry and the release of damage-associated molecular patterns (DAMPs) [12,19]. Lipid peroxidation within meibomian gland secretions, particularly evident in lupus-associated dry eye, alters lipid composition and destabilizes the tear film, contributing to secondary evaporative dysfunction [1,3,19]. Importantly, the contribution of oxidative stress differs across autoimmune entities. In SLE and pSS, oxidative and mitochondrial alterations have been most consistently documented in lacrimal and meibomian gland epithelia through clinical tear analysis, meibography, and experimental models [1,3,19]. In contrast, in OCP, immune-mediated basement membrane injury and TGF-β-driven fibrotic remodeling appear to dominate disease progression, with oxidative stress functioning as a secondary modulator rather than a primary driver [10,22]. These observations support partially shared but disease-specific pathogenic trajectories rather than a uniform mechanistic model.

Emerging antioxidant strategies—including plant-derived polyphenols such as resveratrol and curcumin, along with omega-3 fatty acid formulations—have demonstrated the potential to attenuate oxidative stress, downregulate NF-κB and HIF-1α activation, and mitigate ocular surface inflammation [12,19]. Integrating these agents as adjuvants to existing immunomodulatory regimens provides a translational approach to protecting epithelial integrity, preserving glandular function, and restoring redox balance in autoimmune ocular surface disease [10,11,12,19].

### 2.4. Epithelial–Mesenchymal Transition and Fibrotic Remodeling

Chronic ocular surface inflammation progressively remodels tissue architecture, leading to fibrosis, goblet cell loss, and squamous metaplasia [10,22]. A central mechanism underlying this process is epithelial–mesenchymal transition (EMT), during which epithelial cells lose polarity and junctional integrity while acquiring mesenchymal features associated with migration and contractility [8,10,22]. EMT is primarily driven by transforming growth factor-β (TGF-β), interleukin-17 (IL-17), and matrix metalloproteinases (MMPs), which collectively reprogram epithelial gene expression through Smad2/3- and NF-κB-dependent signaling pathways [10,22]. These events underlie conjunctival fibrosis, symblepharon formation, and scarring characteristic of OCP and severe lupus-related keratopathy [6,20,22].

At the molecular level, EMT involves downregulation of epithelial markers, such as E-cadherin, alongside upregulation of mesenchymal markers, including α-smooth muscle actin (α-SMA) and vimentin [10,22]. Cytokine-mediated crosstalk between epithelial cells and stromal fibroblasts promotes extracellular matrix deposition and stromal fibrosis, ultimately impairing corneal transparency and ocular surface elasticity [8,22]. Oxidative stress and complement activation further potentiate EMT signaling via TGF-β/NF-κB interactions, reinforcing the inflammatory–fibrotic loop [8,10].

Recent single-cell transcriptomic analyses have identified distinct fibroblast subpopulations within the autoimmune ocular surface that express profibrotic markers such as COL1A1, POSTN, and FAP [8,23]. These findings delineate the transition from reversible inflammation to irreversible structural remodeling and support EMT and fibrosis modulation as rational therapeutic targets for preserving epithelial homeostasis and visual function [10,22,23].

### 2.5. Microbiome–Immune Interactions on the Ocular Surface

Although the ocular surface harbors a relatively low microbial biomass, its microbiome plays a critical role in maintaining epithelial integrity, immune tolerance, and antimicrobial defense [3,24]. Dysbiosis—an imbalance within this delicate microbial ecosystem—has been increasingly implicated in autoimmune ocular surface disease, paralleling the immune–microbial disruptions observed in the gut and skin microbiota of patients with systemic autoimmune disorders such as SLE and pSS [3,24]. Metagenomic and 16S rRNA sequencing studies in autoimmune dry eye have demonstrated a reduction in commensal *Corynebacterium* and *Staphylococcus epidermidis* species and a relative expansion of proinflammatory *Demodex*-associated bacteria and other pathogenic taxa [3,19,24].

Microbial dysbiosis activates pattern-recognition receptors, notably toll-like receptors (TLR2 and TLR4), on epithelial and dendritic cells, triggering MyD88- and NF-κB-mediated signaling cascades that amplify local cytokine release [10,24]. This process reinforces Th17-driven responses and sustains chronic ocular surface inflammation. Furthermore, microbial metabolites can influence epithelial differentiation and mucin secretion, linking metabolic signaling with immune regulation and barrier maintenance [3,10,24].

Conversely, restoration of microbial homeostasis—through probiotic supplementation, prebiotic modulation, or selective antibiotic intervention—has shown preliminary success in reducing ocular surface inflammation and improving tear film stability [19,24]. These data collectively position the ocular microbiome as both a molecular biomarker and a promising therapeutic target in autoimmune-mediated ocular surface disease [3,10,19].

### 2.6. Molecular Biomarkers and Genomic Signatures

Advances in multi-omics technologies—encompassing proteomics, transcriptomics, and metabolomics—have profoundly expanded insight into autoimmune ocular surface disease by enabling quantitative molecular profiling of tears and epithelial tissues [4,11,13]. Tear proteomic analyses in patients with SLE and rheumatoid arthritis (RA) have revealed elevated levels of IL-17, MMP-9, and other proinflammatory mediators, along with reduced concentrations of protective proteins such as lactoferrin and lipocalin-1, indicating both epithelial injury and compromised antimicrobial defense [4,11]. In parallel, multi-omics studies in pSS have identified lipidomic alterations, particularly phosphatidylserine PS(36:1), as potential modulators of local immune activity [10,13].

At the genomic level, pyroptosis-related genes—including *NLRP3*, *CASP1*, and *GSDMD*—have been recognized as central regulators of epithelial cell death and chronic ocular surface inflammation in pSS [10,14]. Integrative bioinformatics approaches combining transcriptomic, proteomic, and clinical data have enabled molecular endotyping of disease, facilitating precise patient stratification and personalized therapeutic algorithms [8,14]. The convergence of AI-driven analytics with tear proteomics and metabolomic “liquid biopsy” platforms offers a translational framework for early detection, disease monitoring, and individualized therapy optimization in autoimmune ocular surface disorders [8,10,13,14].

### 2.7. Conceptual Integration of Pathogenic Mechanisms

Although cytokine signaling, complement activation, oxidative stress, epithelial–mesenchymal transition, and microbiome alterations are often described as discrete pathogenic processes, available evidence supports their dynamic and interdependent interaction during autoimmune ocular surface disease progression [5,8,10,11,22]. Early systemic or gland-centric immune activation initiates cytokine- and complement-mediated signaling that disrupts epithelial homeostasis and barrier integrity [4,5,8,15]. This epithelial stress amplifies local oxidative imbalance and innate immune activation, further sustaining inflammatory signaling at the ocular surface [10,11,12,19]. Persistent inflammation promotes EMT-driven fibrotic remodeling of conjunctival and limbal niches, which progressively impairs epithelial regeneration and glandular function [8,10,22,23]. Concurrently, alterations in the ocular microbiome may exacerbate innate immune signaling and perpetuate epithelial dysfunction, reinforcing a self-sustaining pathogenic loop [3,10,19,24]. Together, these mechanisms form a temporal and spatial continuum linking immune activation to epithelial failure, fibrosis, and regenerative insufficiency rather than acting as isolated pathogenic events.

### 2.8. Section Summary

In summary, autoimmune-mediated ocular surface damage arises from a network of interdependent mechanisms encompassing cytokine-driven inflammation, autoantibody- and complement-mediated injury, oxidative stress, and fibrotic epithelial remodeling. Collectively, available evidence indicates that these mechanisms constitute non-equivalent yet functionally interconnected components of autoimmune ocular surface pathology [8,10,11,22]. These interactions define a dynamic pathogenic continuum linking immune activation, epithelial dysfunction, fibrotic remodeling, and progressive regenerative failure. Oxidative stress appears to function primarily as an amplifying and perpetuating factor within inflammatory microenvironments rather than as a singular unifying driver across all autoimmune ocular surface diseases [8,10,22]. Advances in omics-based research have defined distinct molecular signatures that enhance mechanistic understanding while providing measurable biomarkers for diagnostic precision and therapeutic monitoring [6,11,13,14]. Targeting these molecular nodes offers a rational path toward restoring epithelial integrity and immune homeostasis at the ocular surface—an essential goal in the evolving management of autoimmune ocular surface disease [19,24].

## 3. Autoimmune Disorders Affecting the Ocular Surface

Autoimmune diseases affecting the ocular surface constitute a heterogeneous group of disorders in which dysregulated immune responses damage epithelial tissues, sustain chronic inflammation, and progressively remodel ocular surface architecture [4,11]. Clinically, manifestations range from mild KCS to severe cicatricial conjunctivitis and corneal ulceration. Despite overlapping inflammatory mediators and downstream tissue responses, autoimmune diseases affecting the ocular surface differ substantially in their primary immune targets, anatomical focus, and pathogenic hierarchy. These distinctions have direct implications for disease progression, therapeutic responsiveness, and surgical risk, underscoring the importance of disease-specific rather than purely phenotype-based classification [5,11,22].

While SLE, pSS, and OCP are examined in greater mechanistic depth due to the extent of available molecular evidence, the remaining autoimmune conditions discussed in this section are presented as illustrative examples highlighting shared or contrasting patterns of ocular surface involvement rather than as comprehensive mechanistic analyses [4,5,11,22].

For clarity, key inflammatory pathways such as IL-17, type I interferon signaling, and NF-κB activation are referenced in this section primarily to contextualize disease-specific manifestations, while their detailed molecular mechanisms are addressed comprehensively in Section 2.

Accordingly, this section provides a clinico-molecular overview of the principal autoimmune diseases impacting the ocular surface, including SLE, pSS, OCP, TAO, and related systemic connective tissue disorders, emphasizing their distinct immunopathogenic frameworks while highlighting selectively overlapping molecular pathways relevant to ocular surface damage.

### 3.1. Systemic Lupus Erythematosus

Ocular involvement in SLE reflects the systemic nature of immune dysregulation, as autoantibody formation, immune-complex deposition, and complement activation collectively induce inflammatory injury in multiple tissues, including the ocular surface [4,15,25]. The most prevalent ocular features are keratoconjunctivitis sicca, tear film instability, and meibomian gland dysfunction [1,2,7].

Pathophysiologically, SLE-related dry eye extends beyond aqueous deficiency. Immune-mediated destruction of lacrimal acinar cells and altered meibum composition contribute to both aqueous-deficient and evaporative forms [1,3,7]. Immune-complex deposition and type I interferon (IFN-I) pathway activation provoke endothelial and epithelial damage. IFN-α/β enhances antigen presentation through MHC II upregulation, promotes B-cell differentiation, and stimulates IL-6 and TNF-α secretion [10]. These cytokines converge on NF-κB and JAK/STAT signaling in epithelial cells, driving MMP-9 release, oxidative stress, and basement-membrane degradation [4,11,15].

Clinically, SLE patients present with punctate epithelial erosions, filamentary keratitis, and reduced tear break-up time [2,3]. High-resolution meibography reveals gland dropout and lipid-secretion changes [1,26]. Importantly, ocular inflammation may occur independently of systemic disease activity, indicating local immune amplification within adnexal tissues [7]. These abnormalities significantly affect visual comfort and quality of life, supporting early ophthalmologic evaluation [26].

Biologic therapies—belimumab (anti-BAFF) and anifrolumab (anti-IFNAR1)—improve systemic control and may indirectly mitigate ocular inflammation via cytokine modulation [4,15]. However, ocular-specific efficacy data remain limited, emphasizing the need for trials assessing their potential to restore ocular immune homeostasis and guide topical immunomodulation strategies.

### 3.2. Primary Sjögren’s Syndrome

In pSS, lymphocytic infiltration of lacrimal and salivary glands drives secretory dysfunction and chronic ocular surface desiccation, making it the prototype autoimmune exocrinopathy and a major global cause of severe dry eye. Within the lacrimal glands, infiltrating CD4^+^ T and B cells create a cytokine milieu enriched in IL-17, IL-6, BAFF, and IFN-γ, perpetuating acinar destruction and epithelial atrophy [9,13,14].

Recent multi-omics studies reveal molecular heterogeneity in pSS. Spatial metabolomic profiling has identified PS(36:1) enrichment as a key immunometabolic mediator and therapeutic target [13]. Transcriptomic analyses highlight pyroptosis-related genes (*CASP1*, *NLRP3*, *GSDMD*) as regulators of epithelial cell death and chronic inflammation [14]. Experimental CD8^+^ T-cell depletion leads to compensatory Tfh/Tph proliferation, increasing autoantibody production and accelerating glandular injury [27]. Together, these findings link inflammatory, metabolic, and cell-death pathways in pSS pathogenesis.

Clinically, pSS manifests as severe aqueous-deficient dry eye with conjunctival hyperemia, corneal staining, and recurrent epithelial defects [16,28]. Chronic inflammation induces acinar atrophy and fibrosis, causing irreversible gland failure. Tear proteomics reveal MMP-9 and S100A8/A9 upregulation, correlating with epithelial damage [11,28]. Small-fiber neuropathy may exacerbate discomfort and impair reflex tearing [23].

Emerging treatments such as iguratimod—an NF-κB inhibitor—have shown efficacy in reducing ocular and systemic inflammation [16]. MSC-based therapy provides immunomodulatory and regenerative potential for lacrimal gland restoration [17]. These innovations represent a shift from palliative lubrication toward immune-targeted and regenerative strategies for structural and functional recovery in pSS-related ocular disease.

### 3.3. Ocular Cicatricial Pemphigoid

OCP is a chronic, sight-threatening autoimmune disorder mediated by autoantibodies targeting the epithelial basement membrane zone (BMZ). The principal autoantigens—BP180 (type XVII collagen) and laminin-332—are hemidesmosomal components maintaining epithelial adhesion. Their immune recognition activates complement and inflammatory cell recruitment [20,22]. Sustained inflammation leads to subepithelial fibrosis, symblepharon formation, and forniceal shortening, progressing to ankyloblepharon and vision loss if untreated.

Molecularly, OCP exhibits pronounced Th17 and TGF-β activation linking inflammation with fibrosis. Elevated IL-17A, IL-6, and MMP-9 levels in conjunctival tissue drive EMT and fibroblast activation [10]. Analogous to SLE and systemic sclerosis, OCP-associated fibroblasts express α-SMA and collagen I, acquiring a profibrotic phenotype [5,8].

Therapeutic advances include off-label rituximab and JAK-inhibitor use, targeting B-cell and cytokine-driven inflammation. Surgically, amniotic membrane grafts combined with systemic immunosuppression halt scarring and restore epithelial stability [18,29]. OCP thus exemplifies the autoimmune fibrotic continuum—progressing from immune activation to irreversible scarring—and serves as a translational model for mucosal barrier fibrosis.

### 3.4. Thyroid-Associated Ophthalmopathy

Although classically orbital, TAO profoundly affects the ocular surface. Autoimmune activation of orbital fibroblasts expressing TSHR and IGF-1R triggers PI3K/Akt and MAPK signaling, promoting fibroblast proliferation, adipogenesis, and matrix deposition. Activated fibroblasts release IL-1β, IL-6, and TNF-α, recruiting T cells and macrophages that amplify inflammation [30,31]. This cytokine milieu destabilizes the tear film and epithelial barrier, leading to exposure keratopathy and evaporative dry eye [30].

Oxidative stress is a central co-driver of TAO pathology. Mitochondrial dysfunction in orbital fibroblasts enhances ROS generation, lipid peroxidation, and NF-κB/HIF-1α activation, linking oxidative injury with fibrosis and tissue remodeling [30,31]. This damage extends to adnexal glands, contributing to meibomian gland dysfunction and tear instability.

Recent studies emphasize IL-17/TGF-β-mediated crosstalk between immune, fibroblast, and epithelial compartments. This promotes fibroblast-to-myofibroblast transition expressing α-SMA and collagen I, paralleling EMT in other autoimmune ocular diseases [8,10,30].

Teprotumumab (anti-IGF-1R) has emerged as a transformative therapy, reducing inflammation and proptosis while improving ocular surface stability [30,31]. Selenium supplementation mitigates ROS-induced damage and improves tear film parameters [30,31]. Conventional management remains multidisciplinary—local lubrication, corticosteroids, and immunosuppression—with growing interest in JAK inhibitors and MSC therapy for chronic inflammation and fibrosis modulation.

### 3.5. Other Autoimmune Entities Affecting the Ocular Surface

Beyond SLE, pSS, OCP, and TAO, several systemic autoimmune disorders—including connective-tissue diseases—compromise ocular surface integrity through immune-mediated vascular injury, epithelial atrophy, and chronic inflammatory signaling. Systemic sclerosis induces microangiopathy, vascular fibrosis, and epithelial atrophy secondary to immune-mediated ischemia, resulting in tear film instability and conjunctival dryness [26,32]. Dermatomyositis may produce periocular inflammation and exposure keratopathy, while granulomatosis with polyangiitis can cause peripheral ulcerative keratitis and scleritis through ANCA-mediated vasculitis. Chronic inflammation, oxidative stress, and cytokine imbalance converge on shared injury pathways across these conditions [8,10,22].

Autoimmune thyroiditis (Hashimoto’s disease) has also been associated with ocular surface alterations, including tear film instability and epithelial disruption, even in euthyroid states [30]. These findings underscore that ocular surface involvement extends beyond classical autoimmune syndromes.

Early detection of subclinical ocular surface disease is vital in systemic autoimmunity. Noninvasive tools—tear osmolarity, interferometry, and meibography—enable early glandular assessment and guide prompt immunomodulatory therapy [24,33,34]. Such interdisciplinary collaboration between rheumatology and ophthalmology, integrating molecular tear biomarkers into systemic disease management, may facilitate individualized therapeutic strategies [24,34].

### 3.6. Section Summary

Autoimmune diseases damage the ocular surface through mechanisms that differ in primary immune targets yet partially overlap at the level of inflammatory and fibrotic pathways. SLE and pSS exemplify cytokine- and autoantibody-mediated epithelial inflammation, whereas OCP and systemic sclerosis represent fibroinflammatory extremes driven by TGF-β signaling and matrix remodeling. TAO and endocrine autoimmune disorders highlight systemic–local immune–epithelial crosstalk underlying ocular pathology.

Collectively, these diseases illustrate a continuum from immune activation to fibrotic remodeling, unified by cytokine imbalance, oxidative stress, and epithelial–mesenchymal transition. Deciphering these pathways is essential for developing targeted, personalized therapies that preserve ocular surface homeostasis and vision.

A concise overview of the principal molecular mechanisms, ocular manifestations, and therapeutic strategies discussed in this section is summarized in Table 1.

## 4. Pharmacologic and Regenerative Therapies

Managing autoimmune ocular surface disease requires a multimodal, mechanism-oriented therapeutic approach that targets both systemic immune dysregulation and localized epithelial injury. Over the past decade, treatment paradigms have shifted from broad immunosuppression toward precision molecular and regenerative approaches designed to restore ocular surface homeostasis and preserve visual function [4,10,15]. These emerging modalities integrate systemic biologics, topical immunomodulators, and cell- or biomaterial-based regenerative techniques, reflecting the intersection of immunology, molecular pharmacology, and tissue engineering in ophthalmic care [6,10]. For clarity, the therapeutic approaches discussed below are presented along a translational continuum, distinguishing established clinical interventions from emerging strategies supported primarily by preclinical or early-phase evidence [4,6,10,15,16,19].

This section reviews current pharmacologic, regenerative, and surgical interventions, with emphasis on molecular mechanisms, clinical efficacy, and translational relevance in autoimmune ocular surface disease.

### 4.1. Conventional Immunosuppressants and Anti-Inflammatory Agents

Historically, autoimmune ocular surface diseases have been managed through systemic and topical immunosuppression aimed at controlling inflammation and preventing irreversible scarring. Systemic corticosteroids remain the first-line agents for acute exacerbations of SLE, pSS, and OCP due to their potent inhibition of the NF-κB and AP-1 pathways, reducing IL-1β, IL-6, and TNF-α expression and leukocyte infiltration [4,9,15].

Chronic corticosteroid use, however, carries significant ocular and systemic risks, including cataract formation, elevated intraocular pressure, and opportunistic infections [15]. Consequently, steroid-sparing immunosuppressants—such as azathioprine, mycophenolate mofetil, and cyclophosphamide—have become valuable adjuncts, particularly in OCP and severe SLE-related ocular manifestations [5,20].

Topical cyclosporine A (CsA) revolutionized ocular immunomodulation through selective calcineurin inhibition, thereby blocking T-cell activation. CsA enhances tear production, decreases lymphocytic infiltration in lacrimal glands, and restores goblet-cell density, improving tear film stability and epithelial integrity [4,6]. Similarly, lifitegrast, an LFA-1 antagonist, prevents T-cell adhesion to ICAM-1 on epithelial cells, reducing inflammation in both autoimmune dry eye and idiopathic dry eye disease (DED) [16].

Advances in ocular drug delivery—such as nanoemulsions, liposomes, and hydrogel-based systems—have improved CsA bioavailability and tolerability [19]. Collectively, these agents remain essential for treating autoimmune ocular surface disease and form the pharmacologic backbone for integration with emerging biologic and regenerative strategies.

### 4.2. Targeted Biologic Therapies

Advances in translational immunology have led to the development of biologics that selectively modulate immune pathways central to autoimmune ocular surface injury. Among these, the BAFF/BLyS axis—critical for B-cell survival and autoantibody production—represents a key therapeutic target in both SLE and pSS. Belimumab, a monoclonal anti-BAFF antibody, has shown efficacy in reducing systemic disease activity while improving tear secretion and ocular comfort [4,15].

Similarly, blockade of the type I interferon receptor (IFNAR1) with anifrolumab attenuates IFN-driven gene expression and downstream inflammation, offering systemic-to-ocular benefit by mitigating IFN-induced epithelial apoptosis and tear film instability [15,35].

In pSS, Th17 cytokines (IL-17, IL-22) act synergistically with BAFF to sustain glandular inflammation. Targeting this axis using monoclonal antibodies such as secukinumab (anti–IL-17A) or telitacicept (dual BAFF/APRIL inhibitor) holds potential for restoring immune tolerance and lacrimal gland function [9,13,16].

The JAK/STAT cascade, a major mediator of cytokine signaling, represents another promising therapeutic target. Baricitinib (JAK1/2 inhibitor) and deucravacitinib (TYK2 inhibitor) suppress IFN and IL-6 signaling implicated in epithelial injury [10]. Preliminary clinical evidence indicates improved tear stability and ocular staining among patients receiving JAK inhibitors, though ophthalmic-specific trials are limited.

Beyond these, investigational biologics targeting CD38 and CD40L pathways are being evaluated for refractory autoimmune ocular surface disease [4,15]. Collectively, these biologics mark a paradigm shift toward precision immunomodulation, bridging systemic immune regulation with localized ocular protection.

### 4.3. Topical and Localized Immunomodulation

Building upon conventional topical immunosuppressants, recent research has focused on optimizing local drug delivery to achieve sustained immunomodulation with minimal systemic absorption. Nanoformulated and liposomal carriers enhance corneal penetration, prolong drug residence, and improve patient tolerance in chronic autoimmune dry eye [9,19].

Among novel agents, sirolimus (rapamycin) eye drops—an mTOR inhibitor—demonstrate potent anti-inflammatory and antifibrotic effects by reducing T-cell activation and conjunctival scarring in experimental autoimmune models [16,19]. These formulations complement lifitegrast and CsA, expanding the arsenal of localized immune regulation.

Autologous serum and platelet-rich plasma (PRP) eye drops deliver bioactive mediators such as epidermal growth factor, TGF-β, and fibronectin, promoting epithelial regeneration and dampening local immune activity [19]. PRP therapy enhances epithelial healing, tear stability, and comfort by restoring trophic signaling and reducing inflammation.

Emerging sustained-release systems—hydrogel implants, nanocarriers, and biodegradable inserts—offer controlled, long-acting delivery of immunomodulators [19,36]. These platforms address short ocular residence and poor drug stability, representing a critical step toward precision-targeted local immunotherapy.

### 4.4. Regenerative and Cell-Based Therapies

Regenerative medicine represents a transformative frontier in autoimmune ocular surface therapy. Unlike traditional anti-inflammatory approaches, regenerative strategies focus on restoring epithelial structure and rebalancing local immune responses through molecular and cellular repair mechanisms.

MSC therapy exemplifies this paradigm, as MSCs secrete IL-10, TGF-β, and hepatocyte growth factor (HGF), promoting epithelial regeneration and macrophage polarization [16,17]. Clinical studies report improved Schirmer scores, reduced corneal staining, and enhanced comfort in refractory autoimmune dry eye [17,36].

Advances in induced pluripotent stem cells (iPSCs) now enable differentiation into corneal epithelial and retinal ganglion-like lineages, offering dual regenerative potential from a single therapeutic source [37]. This dual-lineage system holds promise for reconstructing damaged ocular surfaces and restoring glandular function in autoimmune disease.

Amniotic membrane transplantation (AMT) remains a cornerstone of regenerative ocular surgery. The membrane’s anti-inflammatory cytokines, growth factors, and ECM components promote epithelial repair and inhibit fibroblast proliferation, aiding recovery in OCP, pSS-related keratopathy, and persistent epithelial defects [18,29].

Next-generation biomaterials, including nanostructured hydrogels, bioengineered scaffolds, and 3D-printed corneal matrices, are expanding the potential of regenerative ophthalmology [18,19,29]. These constructs provide controlled delivery of trophic and anti-inflammatory factors, serving as dynamic microenvironments that guide epithelial migration and enhance cell survival [19,29,37]. By integrating cellular therapy with smart biomaterials, regenerative surgery is shifting from passive tissue replacement toward active bioinductive repair, bridging molecular immunology and clinical ophthalmic reconstruction.

### 4.5. Plant-Derived and Antioxidant Therapies

Beyond synthetic and biologic agents, plant-derived antioxidants offer complementary strategies for mitigating oxidative and inflammatory stress in autoimmune ocular surface disease. Polyphenols, carotenoids, and flavonoids derived from medicinal plants exert potent free-radical scavenging effects and downregulate IL-6, COX-2, and MMP-9, thereby reducing oxidative tissue injury [12,21].

Preclinical models demonstrate that curcumin, quercetin, and resveratrol alleviate ROS-induced apoptosis in corneal epithelial cells by activating the Nrf2/HO-1 pathway and inhibiting NF-κB-mediated inflammation [12]. These compounds stabilize mitochondrial function and restore redox balance, protecting ocular tissues from chronic oxidative stress.

Emerging plant-based eye drops, topical gels, and nutraceutical supplements are under evaluation as adjunctive therapies in autoimmune dry eye and chronic epithelial inflammation. Their integration into multimodal regimens may enhance epithelial resilience, lower oxidative burden, and promote long-term ocular surface stability [12,21].

### 4.6. Ocular Safety, Monitoring, and Translational Considerations

As therapeutic options expand, ongoing evaluation of ocular safety and systemic tolerability becomes paramount. Hydroxychloroquine (HCQ), a cornerstone of SLE therapy, is associated with cumulative dose-dependent retinal toxicity. In contrast, newer biologics—belimumab and anifrolumab—have not shown significant ocular adverse effects to date, though long-term data remain essential [15].

Advanced imaging tools such as optical coherence tomography (OCT) and multifocal electroretinography (mfERG) facilitate early detection of subclinical retinal changes, ensuring timely intervention [15,38]. Incorporating standardized ophthalmic screening into rheumatologic protocols enhances patient safety and therapeutic precision.

Both systemic and local immunosuppression can predispose to opportunistic infections—herpetic keratitis, fungal conjunctivitis, or bacterial superinfections—especially in patients with barrier dysfunction or tear deficiency [5]. Prophylactic antimicrobial strategies and routine ocular monitoring are therefore recommended.

Emerging translational tools, including tear proteomics and AI-based imaging analytics, now allow real-time assessment of cytokine and MMP profiles, correlating molecular activity with clinical outcomes [8,24]. These approaches embody the transition toward precision medicine, enabling individualized monitoring and adaptive therapy in autoimmune ocular surface disease.

### 4.7. Section Summary

Therapeutic strategies for autoimmune ocular surface disease have shifted from empirical immunosuppression toward mechanism-driven precision therapy and regenerative medicine. Conventional agents remain indispensable for acute inflammation control, while biologics, JAK/STAT inhibitors, and MSC-based approaches address the molecular basis of chronic inflammation and degeneration [6,15,16,19].

Integration of bioengineered scaffolds, gene-based delivery systems, and antioxidant formulations represents the next frontier in ocular surface restoration. Concurrently, rigorous safety monitoring and AI-assisted translational tools are redefining personalized care. Collectively, these therapeutic modalities form a continuum of innovation aimed at restoring epithelial integrity, comfort, and long-term immune equilibrium [8,12,19,24]. The therapeutic continuum across conventional, targeted, regenerative, and adjunctive approaches is summarized in Table 2.

## 5. Surgical and Interventional Management

Surgery remains essential for visual rehabilitation and structural restoration in autoimmune ocular surface disease, particularly when pharmacologic therapy fails to control inflammation or reverse tissue damage [18,20]. Recent advances in microsurgery, biomaterial engineering, and regenerative transplantation have significantly improved outcomes in cicatricial, keratopathic, and severe tear film-deficient conditions secondary to systemic autoimmunity [18,29,39].

Modern ocular surface surgery extends beyond mechanical reconstruction by integrating immunomodulation, bioactive scaffolds, and regenerative medicine principles [17,18,29]. This paradigm emphasizes biologically active repair—aimed not only at restoring anatomy but also at re-establishing epithelial integrity, tear film stability, and immune equilibrium [15].

This section outlines key interventional and reconstructive strategies in autoimmune ocular surface disease, focusing on their molecular rationale, clinical outcomes, and synergy with pharmacologic and regenerative therapies.

### 5.1. Principles of Surgical Management in Autoimmune Ocular Surface Disease

Autoimmune-mediated injury often destabilizes the epithelium, promotes conjunctival fibrosis, and alters eyelid position, thereby necessitating staged surgical intervention [18,20]. The primary surgical objectives are to restore ocular surface continuity, normalize eyelid anatomy and blink mechanics, and reestablish a stable tear film supporting epithelial regeneration and vision [5,29].

Active inflammation must be controlled before surgery. Operating in an immunologically active field increases the risk of postoperative fibrosis, graft rejection, and delayed epithelialization [18,20,29]. Comprehensive perioperative immunosuppression—typically involving corticosteroids, calcineurin inhibitors, or biologics such as rituximab or JAK inhibitors—is therefore essential [6,10,15].

Surgical trauma itself may amplify immune activation by releasing DAMPs, triggering IL-1β, IL-6, and TGF-β production, and promoting fibrosis [10]. Consequently, perioperative immune control and regenerative adjuvants have become integral to modern surgical planning and ensuring long-term stability and improved graft survival.

### 5.2. Amniotic Membrane Transplantation

AMT is a key regenerative intervention in autoimmune ocular surface reconstruction, particularly in OCP, pSS-related keratopathy, and chronic epithelial defects in SLE. The membrane functions as a bioactive scaffold that supports epithelial migration, suppresses inflammation, and inhibits fibroblast proliferation through TGF-β inhibition and IL-10 and EGF release [10,18,20].

Cryopreserved AMT preserves native ECM architecture and growth factors, offering superior biocompatibility compared to dehydrated membranes [18,29]. Clinical studies report improved epithelial closure, reduced discomfort, and enhanced visual recovery following AMT in autoimmune dry eye and cicatricial disease unresponsive to pharmacologic therapy [18,29].

At the molecular level, AMT promotes macrophage polarization toward the M2 phenotype, suppresses TGF-β-driven EMT, and reduces IL-1β and MMP-9 expression, thereby limiting conjunctival fibrosis and enhancing graft survival [10,18,20]. Early integration of AMT—combined with systemic or biologic immunomodulation—exemplifies regenerative immunosurgery, where inflammation control and tissue regeneration act synergistically to restore durable ocular surface stability [18,29].

### 5.3. Lid and Conjunctival Reconstruction in Cicatricial Disease

Autoimmune cicatricial disorders such as OCP and chronic lupus blepharitis often cause severe eyelid malpositions—including entropion, trichiasis, and symblepharon—that perpetuate mechanical injury. Anterior lamellar recession and marginal rotation, with or without tarsal fracture, remain the most effective surgical techniques for cicatricial entropion [29].

Marginal rotation with tarsal fracture provides superior eyelid alignment, improving blink dynamics and reducing postoperative dry eye symptoms by restoring lid margin position and meibomian gland function [1,2,29].

When conjunctival scarring is extensive, mucous membrane grafts from buccal or nasal mucosa can resurface the fornix and reconstruct the conjunctival sac. Combining mucosal grafts with AMT and systemic immunosuppression yields optimal outcomes in OCP, minimizing recurrence and preventing new adhesions [18,29]. Emerging bioengineered conjunctival equivalents derived from autologous oral epithelium or allogenic stem cells show reduced immunogenicity and superior integration, representing a promising future for reconstructive ocular surgery [37].

### 5.4. Limbal Stem Cell Transplantation and Bioengineered Corneal Reconstruction

Destruction of the limbal stem cell niche is a central event in chronic epithelial breakdown. Limbal stem cell deficiency (LSCD) presents clinically as recurrent erosions, pannus, and persistent keratitis. In autoimmune disease, LSCD arises from chronic cytokine exposure (IL-6, TNF-α, and IL-17) and complement-mediated microangiopathy, leading to loss of epithelial renewal capacity [10,22].

Reconstruction can be achieved via autologous conjunctival limbal transplantation (CLAU), living-related conjunctival–limbal allograft (lr-CLAL), or keratolimbal allograft (KLAL). However, autoimmune activity and sensitization necessitate long-term systemic immunosuppression to maintain graft survival [4,6,20].

Tissue engineering now enables bioengineered corneal scaffolds seeded with stem cells derived from decellularized cornea or collagen-based hydrogels. iPSCs differentiated into corneal epithelial progenitors can generate transparent epithelial layers after transplantation [37].

Dual-lineage iPSC constructs producing both epithelial and retinal ganglion-like cells represent the next generation of integrated ocular regeneration, bridging corneal and neuroretinal repair [6,37].

### 5.5. Keratoprosthesis and Advanced Ocular Surface Reconstruction

In end-stage cases with irreversible opacity or scarring, keratoprosthesis (KPro) offers visual rehabilitation. Modern devices such as Boston KPro I/II and Osteo-Odonto-KPro yield favorable outcomes in autoimmune etiologies, including OCP and Stevens–Johnson syndrome [39].

Successful KPro implantation requires precise preoperative control of inflammation and tear film restoration, as persistent immune activity increases the risk of corneal melt, extrusion, and endophthalmitis [6,39]. Incorporation of cryopreserved AMT as an interface enhances epithelialization and reduces prosthesis-related complications [18,39].

Emerging hybrid designs integrating bioengineered collagen scaffolds with polymeric cores aim to mimic native corneal biomechanics while minimizing immune degradation—illustrating the convergence of biomaterial design and immunoregulation in ocular reconstruction [29,39].

### 5.6. Laser-Assisted and Minimally Invasive Techniques

Laser-based interventions are gaining importance as minimally invasive adjuncts in autoimmune ocular surface management. Fractional CO_2_ laser blepharoplasty, originally aesthetic, has demonstrated therapeutic benefits in improving tear film stability and meibomian gland function in autoimmune patients [40].

Controlled microthermal stimulation remodels periocular collagen, enhances elasticity, and improves blink mechanics while minimizing scarring compared with conventional surgery [40]. Photobiomodulation (PBM) therapy using low-level laser or near-infrared light further reduces IL-6 and IL-8 expression and supports epithelial healing in chronic autoimmune dry eye [12,40].

These modalities exemplify the shift toward biologically responsive interventions that leverage endogenous regenerative mechanisms, offering minimally invasive alternatives to traditional surgery.

### 5.7. Integrative Immunosurgical Paradigm

Contemporary management increasingly integrates medical and surgical approaches within an immunoregenerative framework. Combining systemic biologics, localized immunomodulators, and regenerative grafts defines this paradigm, where molecular control of inflammation precedes structural repair [5].

Preoperative use of biologics such as belimumab or anifrolumab can stabilize inflammation and mitigate perioperative cytokine surges, improving surgical outcomes [6,15]. Similarly, MSC-conditioned media and PRP-enriched biomaterials serve as intraoperative adjuvants to enhance graft adherence and epithelialization [17,19].

Ultimately, successful ocular surface surgery relies not only on technical precision but also on achieving molecular harmony between inflammation control, regenerative activation, and immune tolerance—a triad that defines modern ocular surface surgery in autoimmune disease.

### 5.8. Section Summary

Surgical and interventional management of autoimmune ocular surface disease has evolved from reactive repair to proactive, biology-driven reconstruction. Amniotic membrane transplantation, limbal stem cell therapy, and keratoprosthesis represent sequential interventions tailored to disease severity. The integration of stem cell technologies, laser-assisted remodeling, and biomaterial engineering defines a new translational frontier in ocular surgery.

Modern ocular surface reconstruction is no longer purely mechanical—it represents immunoregenerative therapy, uniting immune modulation and tissue regeneration to restore both form and function.

## 6. Translational Perspectives and Future Directions

Over the past decade, research on autoimmune ocular surface disease has moved beyond descriptive clinical observation toward mechanistic insight and translational experimentation [8,13]. Despite significant advances, substantial gaps remain between molecular discovery and clinical application [10,14]. Importantly, many of these advanced approaches remain at preclinical or early translational stages and should be interpreted as future therapeutic directions rather than near-term clinical options.

Closing these gaps will require the integration of multi-omics analytics, artificial intelligence (AI), advanced biomaterials, and personalized immunotherapy. Together, these elements define the emerging paradigm of ocular immunoregeneration, in which immune modulation converges with regenerative ophthalmology [17,34,37,39].

### 6.1. Integrative Multi-Omics and Systems Immunology

The pathogenesis of autoimmune ocular surface disease reflects complex interactions among epithelial, immune, and systemic inflammatory networks. Traditional biomarkers cannot capture this multidimensionality, whereas multi-omics profiling—encompassing genomics, transcriptomics, proteomics, lipidomics, and metabolomics—offers a holistic understanding of disease heterogeneity [5,6,14].

Spatial metabolomic and transcriptomic studies in pSS and SLE have identified distinctive lipidomic alterations, including dysregulation of PS(36:1) and sphingolipid metabolism [13]. These metabolic changes correlate with immune infiltration and may act as checkpoints of ocular immune tolerance. Tear proteomics further highlights reproducible biomarkers such as MMP-9, lipocalin-1, and S100A8/A9, reflecting inflammatory burden and therapeutic response [4,11,24].

When integrated through computational modeling, these datasets enable systems-level mapping of ocular autoimmunity. Machine-learning algorithms have delineated pyroptosis-related clusters (e.g., *NLRP3*, *CASP1*, and *GSDMD*) predictive of severe inflammation and poor therapeutic response [14]. As data resources expand, multi-omics signatures are expected to guide early diagnostics, subtype stratification, and personalized immunotherapy design.

### 6.2. Artificial Intelligence and Digital Ophthalmic Biomarkers

AI and deep learning are transforming the interpretation of ophthalmic imaging. Confocal microscopy, OCT, and meibography datasets analyzed by convolutional neural networks allow automated quantification of epithelial thickness, meibomian morphology, and inflammatory infiltration [24,34].

AI-driven pattern recognition surpasses manual grading in detecting early autoimmune changes such as glandular dropout and tear film instability. Integrating imaging metrics with proteomic or metabolomic data enhances predictive models of disease trajectory and therapeutic efficacy [8,34].

Beyond diagnostics, AI enables teleophthalmology and continuous disease monitoring, linking digital biomarkers with electronic medical records. These systems will underpin precision ocular medicine, uniting molecular analytics with individualized treatment planning [24,41].

### 6.3. Gene- and Cell-Based Therapeutic Frontiers

Next-generation therapeutic strategies aim to intervene at the genetic and cellular levels. CRISPR–Cas9 editing provides a preclinical platform for silencing proinflammatory mediators such as IL-17, BAFF, and NLRP3, potentially achieving durable immune recalibration [8,10].

Stem cell-based therapies continue to redefine ocular regeneration. In preclinical and experimental models, iPSC-derived epithelial progenitors have demonstrated the capacity to reconstruct transparent corneal epithelial layers, while dual-lineage constructs incorporating retinal ganglion-like cells illustrate the conceptual potential for integrated corneal and neuroretinal repair [37,42].

In parallel, MSC-derived exosomes offer a non-cellular alternative to cell therapy. These microvesicles carry regulatory microRNAs and reproduce many of the paracrine and anti-inflammatory effects of MSCs, with lower immunogenic and oncogenic risk [17,19,42]. Collectively, these biotechnologies illustrate the continuum from molecular insight to functional tissue regeneration.

### 6.4. Microbiome-Based Immunomodulation

The ocular microbiome has emerged as a crucial regulator of epithelial and immune balance. Autoimmune dysbiosis—marked by reduced commensal *Corynebacterium and Staphylococcus epidermidis*—activates TLR2/TLR4 signaling and drives chronic inflammation [3,24,43].

Restoration of microbial homeostasis through probiotic supplementation, postbiotic metabolites, or engineered microbiota represents an emerging and largely exploratory therapeutic avenue. Pilot studies suggest that *Lactobacillus*-based probiotics can downregulate IL-17 and improve tear stability in autoimmune dry eye [19,43].

Looking ahead, synthetic biology may enable the design of commensal strains engineered to secrete anti-inflammatory cytokines or antimicrobial peptides directly on the ocular surface—introducing a living, self-regulating therapy uniting local and systemic immune tolerance.

### 6.5. Toward Personalized and Translational Immunotherapy in Autoimmune Ophthalmology

Autoimmune ocular surface disease demonstrates substantial heterogeneity—from cytokine-driven inflammation in SLE to pyroptosis-mediated epithelial loss in pSS [9]. The integration of multi-omics, AI analytics, and immunogenetic profiling enables stratification into molecularly defined subgroups with distinct therapeutic susceptibilities [9,15]. Patients with strong IFN-I signatures may benefit from IFNAR blockade (anifrolumab), whereas BAFF- or IL-17-dominant phenotypes respond to belimumab or secukinumab [4,9,15]. Individuals with oxidative stress-driven profiles may benefit from adjunct antioxidant therapy [12].

Computational decision-support systems are being developed to predict disease trajectories and optimize individualized therapeutic combinations of biologics, MSC-derived exosomes, and bioengineered constructs [17,18,19,29]. These frameworks mark the transition of autoimmune ophthalmology from a reactive to a predictive, preventive, and data-driven discipline [24,34].

Translating these innovations into clinical reality requires addressing ethical and regulatory challenges: long-term gene-editing safety, standardization of stem cell-derived products, and oversight for advanced biomaterial matrices [37]. Similarly, AI-based diagnostics must comply with data privacy, algorithmic transparency, and equitable access standards [24,34].

Ultimately, the successful implementation of personalized immunotherapy will depend on multidisciplinary collaboration among immunologists, ophthalmologists, data scientists, and regulatory agencies—balancing innovation with patient safety. Standardized outcome measures integrating tear proteomics, imaging biomarkers, and quality-of-life indices will be essential for cross-study comparability and clinical adoption of next-generation therapies [8,34].

### 6.6. Section Summary

The translational horizon of autoimmune ocular surface research is defined by the convergence of molecular immunology, digital diagnostics, and regenerative biotechnology. Multi-omics analyses and AI-driven imaging are redefining disease taxonomy, while gene editing, cell-based constructs, and microbiome modulation herald a new era of targeted, personalized immunotherapy.

The ultimate goal extends beyond suppressing inflammation—to rebuild immune equilibrium and achieve authentic ocular regeneration, transforming vision science into a discipline of precision immunorepair.

## 7. Conclusions

Autoimmune ocular surface diseases represent one of the most intricate intersections between systemic immune dysregulation and localized tissue pathology. While several emerging strategies discussed in this review remain at early or preclinical stages, distinguishing established therapies from exploratory approaches is essential for realistic translational interpretation. Although these diseases converge clinically on chronic epithelial dysfunction and visual impairment, their underlying pathogenic mechanisms differ in relative contribution and tissue specificity. Shared molecular features should be interpreted as overlapping pathways rather than evidence of a single unifying disease mechanism. This heterogeneity underscores the need to define the molecular and cellular diversity underlying these entities, as understanding their specific immunological microenvironments is essential for designing precise and effective therapies.

Over the past decade, the therapeutic paradigm has shifted from broad immunosuppression toward mechanism-specific, regenerative, and personalized strategies. Conventional agents such as corticosteroids and cyclosporine A remain indispensable for acute inflammatory control but are now complemented by targeted biologics acting on cytokine networks (BAFF, IFN-I, IL-17) and small-molecule inhibitors modulating intracellular cascades such as JAK/STAT and NF-κB. Together, these treatments provide a refined immunomodulatory spectrum that suppresses inflammation while preserving physiological tissue repair.

Concurrently, advances in regenerative medicine have expanded therapeutic horizons beyond pharmacologic control. Mesenchymal- and iPSC-based therapies, amniotic membrane transplantation, and bioengineered corneal scaffolds have demonstrated that structural regeneration and immune modulation can coexist within a unified therapeutic model. This evolution defines the emergence of immunoregenerative ophthalmology—a discipline aiming not merely to inhibit inflammation but to restore immune homeostasis, epithelial integrity, and visual function.

At the molecular frontier, multi-omics technologies and artificial intelligence are reshaping the conceptual and diagnostic framework of autoimmune ocular surface disease. Integrated genomic, proteomic, and metabolomic analyses enable precise molecular subtyping, while AI-assisted imaging provides real-time, objective assessment of ocular inflammation, tear film stability, and meibomian gland architecture. The convergence of these tools is establishing the foundation for precision ophthalmology, in which individualized molecular signatures guide therapeutic choices, dosage optimization, and prognostic modeling.

Equally transformative are emerging gene- and microbiome-based interventions. The ability to modulate inflammatory gene expression through CRISPR–Cas editing, combined with microbiome restoration via targeted probiotics or engineered commensal strains, introduces a new dimension of biologically harmonized therapy. These approaches not only attenuate downstream inflammation but may also recalibrate upstream immune dysfunction responsible for epithelial degeneration.

Despite rapid progress, critical translational and ethical challenges remain. Long-term validation of gene-editing safety, standardization of cell-derived therapeutic products, and robust regulatory frameworks for AI-based diagnostics are essential to ensure clinical reliability and patient safety. The development of universally accepted biomarkers and outcome measures will further enable cross-study comparability and evidence-based clinical translation.

Looking ahead, the most impactful therapies will likely emerge from multidisciplinary integration—linking immunology, regenerative medicine, bioengineering, and computational science. The future of autoimmune ocular surface management will depend not on isolated interventions but on coordinated immune reprogramming and tissue regeneration tailored to each patient’s molecular landscape.

Ultimately, the goal of this evolving field extends beyond preserving vision or relieving symptoms. It aspires to restore ocular immune equilibrium and visual vitality, transforming chronic autoimmune disorders into manageable and potentially reversible diseases. The accelerating synergy between molecular discovery, precision diagnostics, and regenerative innovation suggests that this future is no longer theoretical—it is actively unfolding at the intersection of translational immunology and clinical ophthalmology.

## Figures and Tables

**Table 1:** Summary of key molecular mechanisms, ocular surface manifestations, and therapeutic strategies across major autoimmune diseases affecting the ocular surface.

Disease	Key Molecular Mechanisms	Principal Ocular Manifestations	Emerging/Current Therapeutic Approaches
SLE	IFN-I activation; complement deposition; cytokine storm (IL-6, TNF-α); oxidative stress	KCS, MGD, epithelial erosions, keratopathy	Belimumab, Anifrolumab, Cyclosporine A, Lifitegrast
pSS	Th17/BAFF axis; pyroptosis-related genes (*NLRP3*, *GSDMD*); lipid dysregulation	Severe dry eye, conjunctival staining, fibrosis	Iguratimod, MSC therapy, topical immunomodulators
OCP	Autoantibodies to BP180 and laminin-332; IL-17/TGF-β signaling; EMT activation	Symblepharon, conjunctival scarring, conjunctival fornix shortening	Rituximab, JAK inhibitors, amniotic membrane grafts
TAO	TSHR/IGF-1R activation; oxidative stress; cytokine-mediated tissue remodeling	Exposure keratopathy, dry eye, eyelid retraction	Teprotumumab, selenium supplementation, ocular lubricants
Systemic sclerosis/Other connective tissue diseases	Endothelial fibrosis; vascular ischemia; chronic inflammation; cytokine imbalance	Dry eye, keratitis, conjunctival telangiectasia	Systemic immunosuppression, antioxidants, ocular lubricants

Abbreviations: SLE—systemic lupus erythematosus; pSS—primary Sjögren’s syndrome; OCP—ocular cicatricial pemphigoid; TAO—thyroid-associated ophthalmopathy; KCS—keratoconjunctivitis sicca; MGD—meibomian gland dysfunction; EMT—epithelial–mesenchymal transition; MSC—mesenchymal stem cell.

**Table 2:** Summary of pharmacologic, biologic, regenerative, and adjunctive therapeutic modalities in autoimmune ocular surface disease.

Therapeutic Class	Mechanism of Action	Key Molecular Targets	Representative Agents	Primary Indications	Translational Stage
Conventional Immunosuppressants	Inhibition of NF-κB and AP-1 pathways; suppression of proinflammatory cytokines (IL-1β, TNF-α, IL-6)	NF-κB, IL-6, TNF-α	Corticosteroids, Azathioprine, Cyclosporine A	SLE, OCP, pSS	Established
Targeted Biologic Therapies	BAFF/IFN blockade; JAK/STAT pathway inhibition	BAFF, IFNAR1, JAK1/2, TYK2	Belimumab, Anifrolumab, Baricitinib, Deucravacitinib	SLE, pSS	Clinical use
Topical and Localized Immunomodulators	Local T-cell adhesion inhibition; calcineurin and mTOR blockade	ICAM-1/LFA-1, Calcineurin, mTOR	Lifitegrast, Nanoformulated Cyclosporine A, Sirolimus	Autoimmune DED, pSS	Clinical use
Regenerative and Cell-Based Therapies	Immunomodulation; paracrine signaling; epithelial regeneration	IL-10, TGF-β, microRNAs	Mesenchymal stem cells (MSCs), induced pluripotent stem cells (iPSCs), amniotic membrane transplantation (AMT)	pSS, OCP, SLE	Translational/early clinical
Antioxidant and Plant-Derived Therapies	ROS scavenging; Nrf2/HO-1 activation; NF-κB inhibition	Nrf2, HO-1, NF-κB	Curcumin, Resveratrol, Quercetin, plant extracts	DED, pSS, SLE	Preclinical
Adjunctive/Autologous Therapies	Delivery of epithelial growth factors and anti-inflammatory mediators	EGF, TGF-β, Fibronectin	Platelet-rich plasma (PRP), autologous serum	Severe ocular surface damage, refractory DED	Clinical use

Abbreviations: SLE—systemic lupus erythematosus; pSS—primary Sjögren’s syndrome; OCP—ocular cicatricial pemphigoid; DED—dry eye disease; MSC—mesenchymal stem cell; iPSC—induced pluripotent stem cell; AMT—amniotic membrane transplantation; PRP—platelet-rich plasma; NF-κB—nuclear factor kappa-light-chain-enhancer of activated B cells; JAK—Janus kinase; STAT—signal transducer and activator of transcription; BAFF—B-cell activating factor; IFN—interferon; IFNAR1—interferon alpha receptor 1; mTOR—mammalian target of rapamycin; Nrf2—nuclear factor erythroid 2–related factor 2; HO-1—heme oxygenase-1; ROS—reactive oxygen species; EGF—epidermal growth factor; TGF-β—transforming growth factor beta; ICAM-1—intercellular adhesion molecule 1; LFA-1—lymphocyte function-associated antigen 1; TNF-α—tumor necrosis factor alpha; IL—interleukin.

## Data Availability

No new data were created or analyzed in this study. Data sharing is not applicable to this article.

## Abbreviations

The following abbreviations are used in this manuscript:

AI	Artificial intelligence
AMT	Amniotic membrane transplantation
BAFF	B-cell activating factor
BMZ	Basement membrane zone
CLAU	Conjunctival limbal autograft
CsA	Cyclosporine A
DAMPs	Damage-associated molecular patterns
DED	Dry eye disease
ECM	Extracellular matrix
EGF	Epidermal growth factor
EMT	Epithelial–mesenchymal transition
HGF	Hepatocyte growth factor
HO-1	Heme oxygenase 1
IFN	Interferon
IFNAR1	Interferon alpha receptor 1
IFN-I	Type I interferon
IL	Interleukin
iPSC	Induced pluripotent stem cell
JAK	Janus kinase
KCS	Keratoconjunctivitis sicca
KLAL	Keratolimbal allograft
KPro	Keratoprosthesis
lr-CLAL	Living-related conjunctival–limbal allograft
LFA-1	Lymphocyte function-associated antigen 1
MAPK	Mitogen-activated protein kinase
MGD	Meibomian gland dysfunction
mfERG	Multifocal electroretinography
MMP	Matrix metalloproteinase
MSC	Mesenchymal stem cell
NF-κB	Nuclear factor kappa-light-chain-enhancer of activated B cells
Nrf2	Nuclear factor erythroid 2–related factor 2
OCP	Ocular cicatricial pemphigoid
OCT	Optical coherence tomography
PBM	Photobiomodulation
PRP	Platelet-rich plasma
PS(36:1)	Phosphatidylserine (36:1)
pSS	Primary Sjögren’s syndrome
RA	Rheumatoid arthritis
ROS	Reactive oxygen species
SLE	Systemic lupus erythematosus
TAO	Thyroid-associated ophthalmopathy
TGF-β	Transforming growth factor beta
TNF-α	Tumor necrosis factor alpha
α-SMA	Alpha-smooth muscle actin
